# Cerebral Blood Flow Disorder in Acute Subdural Hematoma and Acute Intraoperative Brain Bulge

**DOI:** 10.3389/fneur.2022.815226

**Published:** 2022-04-01

**Authors:** Liang Xian, Cheng Wang, Liangfeng Wei, Shousen Wang

**Affiliations:** ^1^Fuzong Clinical Medical College of Fujian Medical University, Fuzhou, China; ^2^Department of Neurosurgery, The First Affiliated Hospital of Wannan Medical College, Wuhu, China; ^3^Department of Neurosurgery, 900 Hospital of the Joint Logistics Team, Fuzhou, China

**Keywords:** acute subdural hematoma (ASDH), brain bulge, cerebral blood flow (CBF), laser speckle contrast imaging (LSCI), rat

## Abstract

**Context:**

Acute subdural hematoma (ASDH) has a high incidence and high mortality. During surgery for ASDH, brain tissue sometimes rapidly swells and protrudes into the bone window during or after removal of the hematoma. This phenomenon, known as acute intraoperative brain bulge, progresses rapidly and can cause ischemic necrosis of brain tissue or even mortality. The mechanism of this phenomenon remains unclear.

**Objective:**

To investigate the changes in cerebral surface blood flow during ASDH and acute intraoperative brain bulge in rats.

**Methods:**

Adult male Sprague–Dawley rats were selected to establish an ASDH model, and acute intraoperative brain bulge was induced by late-onset intracranial hematoma. The changes in cerebral surface blood flow during ASDH and acute intraoperative brain bulge were observed with a laser speckle imaging system, and intracranial pressure (ICP) was monitored.

**Results:**

ICP in rats increased significantly after ASDH (*P* < 0.05). The blood perfusion rate (BPR) values of the superior sagittal sinus, collateral vein and artery decreased significantly in rats with subdural hematomas (*P* < 0.05). There was no significant difference between the preoperative and 90-min postoperative BPR values of rats. ICP was significantly increased in rats with acute intraoperative brain bulge (*P* < 0.05) and decreased significantly after the removal of delayed hematomas (*P* < 0.05). The BPR of the superior sagittal sinus, collateral vein and artery decreased significantly during brain bulge (*P* < 0.05). After the removal of delayed hematomas, BPR increased significantly, but it remained significantly different from the values measured before brain bulge (*P* < 0.05).

**Conclusion:**

ASDH may cause not only high intracranial pressure but also cerebral blood circulation disorders. Brain bulge resulting from late-onset intracranial hematoma may aggravate these circulation disorders. If the cause of brain bulge in a given patient is late-onset intracranial hematoma, clinicians should promptly perform surgery to remove the hematoma and relieve circulation disorders, thus preventing more serious complications.

## Introduction

Acute subdural hematoma (ASDH) has a high incidence and high mortality (1). During surgery for ASDH, brain tissue sometimes rapidly swells and protrudes into the bone window during or after removal of the hematoma. This phenomenon, known as acute intraoperative brain bulge, progresses rapidly and can cause ischemic necrosis of brain tissue or even death (2). The mechanism of this phenomenon remains unclear. Many researchers have discussed the classification, prevention and treatment of acute brain bulge during surgery, but most studies to date have been clinical; few laboratory studies have explored this topic. In fact, no animal model of acute intraoperative brain bulge has been reported in any foreign or domestic study.

Due to the serious consequences of acute intraoperative brain bulge, it is necessary to explore the pathogenesis of this phenomenon and possible strategies for its treatment. To date, several theories about the mechanism of acute intraoperative brain bulge have been proposed based on clinical studies. The main suggested mechanisms include late-onset intracranial hematoma, acute diffuse brain swelling, and venous reflux disorder (3–7), but none of these theories can effectively account for all features of the condition. Broadly, the mechanism of acute intraoperative brain bulge may be related to blood circulation (2). The purpose of the current study was to explore this possible mechanism and provide ideas from which to formulate appropriate treatment strategies.

Acute intraoperative brain bulge mainly occurs after ASDH. Therefore, in this study, an ASDH rat model was used to simulate the conditions producing acute intraoperative brain bulge, providing a method to study brain bulge in detail and further understand its mechanism.

In this paper, changes in cerebral blood flow in acute subdural hematoma and acute intraoperative brain bulge were observed in animals, laying a foundation for the study of the mechanism and treatment of acute intraoperative brain bulge.

## Materials and Methods

### Animals and General Preparation

This experiment was approved by the Fujian Medical University Ethics Committee and was in accordance with the Guide for the Care and Use of Laboratory Animals (Institute for Laboratory Animal Research, National Research Council. Washington, DC: National Academy Press, 1996). Twenty-four adult male Sprague–Dawley (SD) rats weighing 350–370 g were randomly divided into 4 groups, designated A, B, C and D, with six rats in each group. The top of the head and right inguinal areas were shaven and disinfected. After anesthesia was induced with 3% isoflurane gas, the rats were fixed on a stereotaxic frame, and anesthesia was maintained with 2% isoflurane. A small animal life monitor (Alcott Biological Co., Ltd., Shanghai, China) was connected to a catheter in the right femoral to monitor blood pressure, and a respiratory probe was used to monitor respiratory rate and tidal volume. Body temperature was maintained at 37.0°C with a heating pad throughout the procedure. A 1.5-cm incision was made in the middle of the scalp.

### Intracranial Pressure Monitoring

Before the subdural hematoma model was made, a craniotomy of approximately 2 mm was drilled at 1.5 mm in front of the coronal suture and 1.5 mm to the left of the sagittal suture. An intracranial pressure probe (Type 82-6631, Johnson & Johnson Inc., USA) was inserted into the brain parenchyma at a depth of 4 mm. The sensor was connected to a Codman intracranial pressure monitor (Johnson & Johnson Inc., New Brunswick, USA).

### Acute Subdural Hematoma Model and Cortical Blood Flow Observation

**Figure 1:**
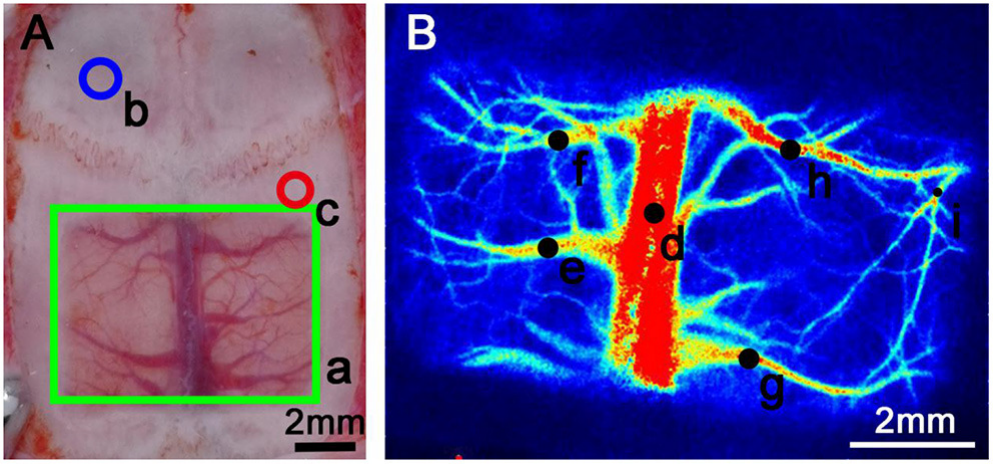
Schematic diagram of transparent bone window preparation and LSCI monitoring of the rat skull. **(A)** a: The green rectangular area is the transparent bone window area, with dimensions of 7 × 5 mm. The skull was polished until transparent, making the underlying blood vessels visible. b: The blue circle marks the area where the intracranial pressure probe was placed into a craniotomy. The center point was 1.5 mm in front of the coronal suture and 1.5 mm to the left of the sagittal suture, with a diameter of 2 mm. c: The red circle marks the area where a craniotomy was created to allow injection into the subdural hematoma. The central point was 1.5 mm behind the coronal suture and 3 mm to the right of the sagittal suture, with a diameter of 1.3 mm. **(B)** The black dots on the figure show the regions of interest (ROIs). d: Area of the superior sagittal sinus observation point. e–h: Collateral venous observation point. i: Arterial observation point. The areas above were selected for monitoring according to the specific experimental procedure because the hematoma formed by the injection would cover other blood vessels in the ipsilateral hemisphere; the actual measurement of the ipsilateral hemisphere was performed only on the selected blood vessels.

In the rats in group A and group B, a 7 × 5 mm bone window between the coronal suture and the lambdoid suture was polished until the skull was transparent and the underlying blood vessels were visible. Group A: A craniotomy was drilled 1.5 mm behind the coronal suture and 3 mm to the right of the sagittal suture with a diameter of 1.3 mm under a surgical microscope. The dura was punctured under a surgical microscope to open a 1-mm-diameter breach. A spindle-shaped 8-G mouse gavage needle (maximum outer diameter 1.4 mm, top minimum outer diameter 1 mm, inner diameter 0.8 mm) was quickly inserted into the bone hole, with the opening of the needle close to the hole in the dura. Then, 0.4 ml of non-heparinized autogenous blood was extracted from the femoral vein with a 1-ml syringe, and the excess air was expelled from the syringe. The autogenous blood was placed in a water bath with a constant temperature of 37.0°C for 3 min to slightly coagulate some of the blood. The syringe was connected to a needle and injected slowly into the subdural space with a microinjection pump over a period of 5 min at a speed of 0.08 ml/min. The needle was removed immediately after the injection, and the craniotomy was sealed with bone wax. Group B (Sham group): This group, which served as a blank control, underwent the same operation as group A except that blood was not injected. Hemodynamic changes were monitored using a laser speckle contrast imaging (LSCI) system (Rayward, Shenzhen, China). Blood flow through the superior sagittal sinus, cortical vein and artery of each rat was observed through a transparent bone window by LSCI (Figure 1). Blood flow velocity and blood vessel diameter were recorded before the operation (before injection) and 5, 30, 60, 90, and 120 min after the operation. All rats were euthanized after the experiment.

### Cortical Blood Flow Observation During Acute Intraoperative Brain Bulge

**Figure 2:**
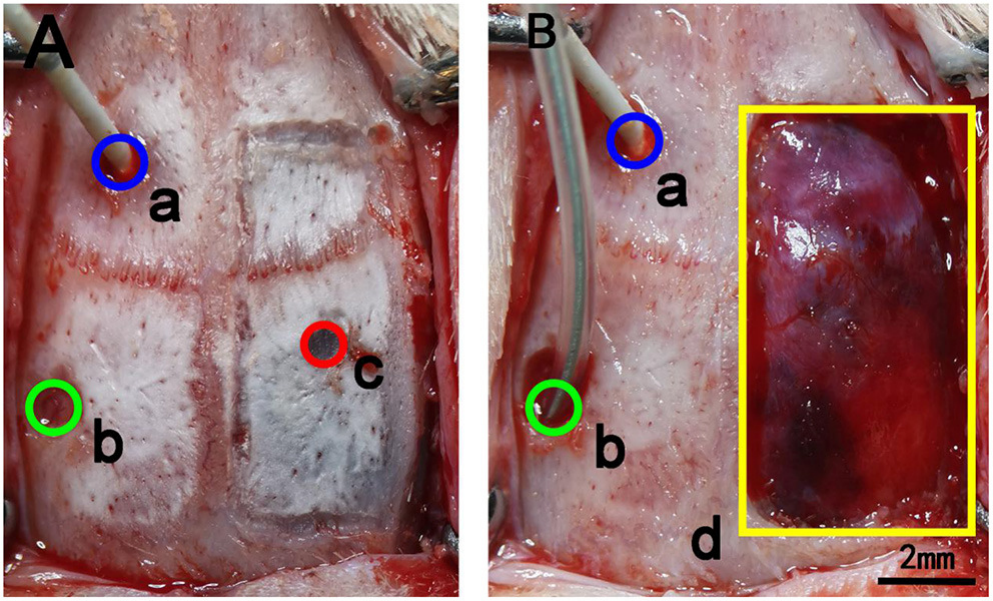
Schematic diagram of the removal of bone flaps in rats. **(A)** a: The blue circle marks the area where the intracranial pressure probe was inserted through a craniotomy. The center point is 1.5 mm in front of the coronal suture and 1.5 mm to the left of the sagittal suture, and the diameter of the circle is 2 mm. b: The green circle marks the craniotomy for balloon placement. The central point is 2 mm behind the coronal suture and 4 mm to the left of the sagittal suture, and the diameter of the opening is 2 mm. c: The red circle marks the site of the injected subdural hematoma. The central point is 1.5 mm behind the coronal suture and 3 mm to the right of the sagittal suture, and the diameter is 1.3 mm. **(B)** The yellow rectangle shows the location of the bone window opened for hematoma removal. The size of the bone window was 10 × 5 mm. In the figure, the bone window has been removed, but the dura is intact.

Rats in groups C and D were selected to establish subdural hematoma models. A bone hole was drilled 3 mm behind the left coronal suture and 5 mm beside the left sagittal suture, with a radius of 2 mm, and the integrity of the lower dura was ensured. After that, a bone flap measuring 10 × 5 mm was removed from the right hematoma injection site of each rat under a surgical microscope (Figure 2). A cross-shaped incision was made in the dura, and the gelatinous hematoma was removed carefully with forceps. Then, the cortex was slowly and repeatedly rinsed with saline. A water-filled balloon was inserted into the left craniotomy and placed in the epidural space. Group C: The blood flow of cortical veins and arteries was observed through the bone window, and the blood flow velocity and blood vessel diameter were recorded immediately after bone flap removal. After that, the balloon was slowly injected with water to 0.1 ml to simulate epidural hematoma. Brain tissue swelling on the side of bone flap removal was observed, bone window protrusion was observed, and the speckle data were recorded. After the balloon had been in place for 10 min, we made another recording of the speckle data again. Then, the balloon was removed, and the speckle data were recorded again; they were recorded one final time 10 min later. In group D (Sham group), the balloon was not injected with water, and the data were recorded immediately after the removal of the bone flap as well as 5 min and 10 min later. The balloon was removed, and the speckles were recorded again 10 min later. All rats were euthanized after the experiment.

### Statistical Analysis

LSCI software was used to select the region of interests (ROIs) and measure the diameters of blood vessels, and the values obtained were used to calculate the average blood flow value of the region (8). To avoid errors caused by diameter changes, the blood perfusion rate (BPR) was calculated as follows (9, 10):


$$\begin{array}{l} \text{BPR}=\text{V}\times \frac{\pi \times {\text{D}}^{2}}{4} \end{array}$$

where *V* = blood flow velocity and *D* = vessel diameter.

All data are expressed as the mean ± standard deviation. SPSS 19.0 was used to process and analyze the data. All data were tested for normality, and all data conformed to a normal distribution. Repeated-measures ANOVA was used to compare BPR at each time point. Repeated-measures ANOVA was also used to compare groups. *P* < 0.05 indicated a statistically significant difference.

## Results

### Blood Flow in Animals With Acute Subdural Hematomas

**Figure 3:**
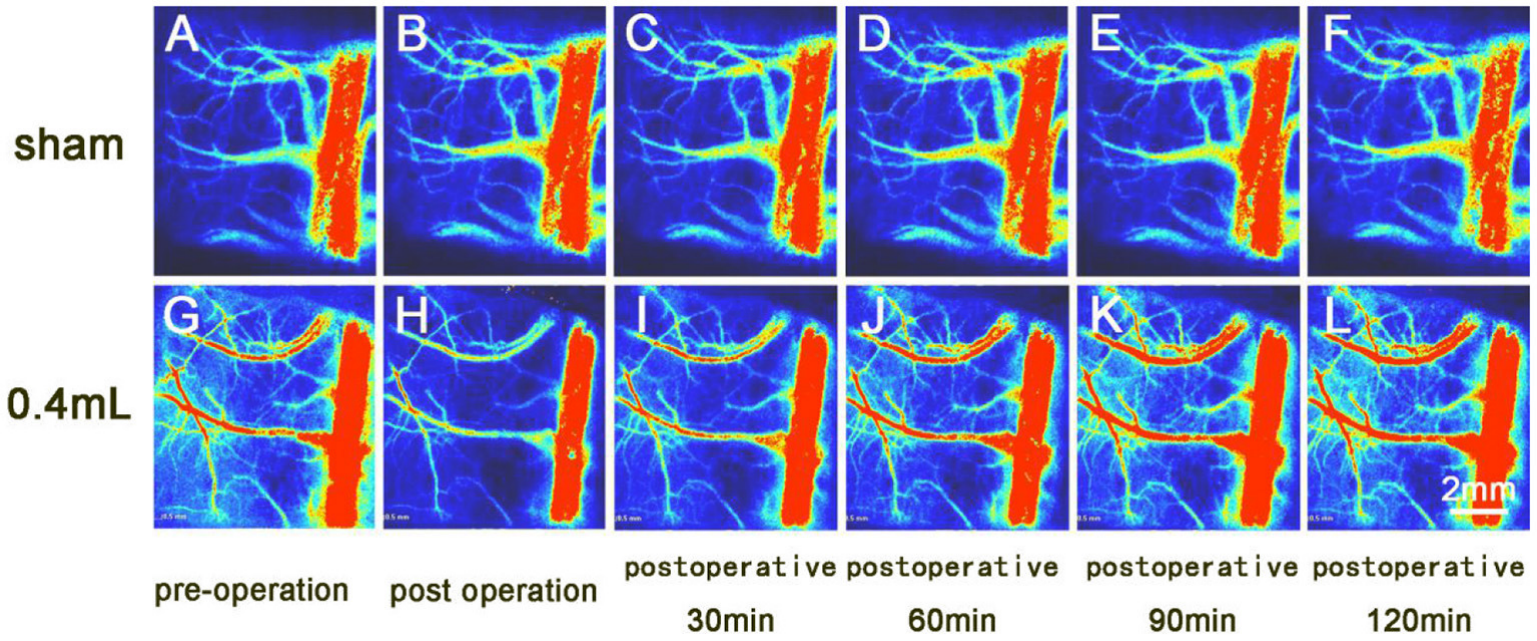
Laser speckle imaging of arterial and venous blood flow on the side opposite the hematoma. **(A–F)** The cerebral surface blood flow of the sham group was essentially unchanged over the 120-min observation period. **(G–L)** The cerebral surface blood flow of the 0.4 ml group decreased after hematoma injection, and the veins and arteries gradually recovered by 90 min after surgery, while the superior sagittal sinus did not recover its preoperative blood flow.

The BPR of the Sham group remained essentially unchanged. The BPR of the two experimental groups decreased after surgery and recovered after 60–90 min. The BPR values of the two experimental groups showed a decreasing trend after surgery but showed a trend toward recovery after 60–90 min (Figure 3). The trend of intracranial pressure in the Sham group was essentially the same as that of BPR, and the intracranial pressure in the two experimental groups increased and then stabilized after surgery.

#### Results From the 0.4 ml Group

##### Preoperative Vascular BPR

There was no significant difference in preoperative BPR at the superior sagittal sinus, vein or artery between the 0.4 ml group and the Sham group.

##### Vascular BPR Within 60 min After Surgery

Five minutes after surgery, vascular BPRs were significantly lower than before surgery (all *P* < 0.001). Compared with the BPR of the Sham group at 5 min, these BPRs were all significantly reduced, with *P* values of 0.002, 0.014 and 0.004, respectively.

The BPRs 30 min after surgery were lower than the preoperative BPRs (*P* < 0.001). Compared with the BPRs of the Sham group at this time point, the differences were statistically significant, with *P* values of 0.003, 0.006 and 0.006.

Sixty minutes after surgery, the BPRs were still significantly lower than before surgery (*P* < 0.05). Compared with the BPRs of the Sham group at this time point, these BPRs were significantly reduced, with *P* values of 0.018, 0.024 and 0.008, respectively.

**Figure 4:**
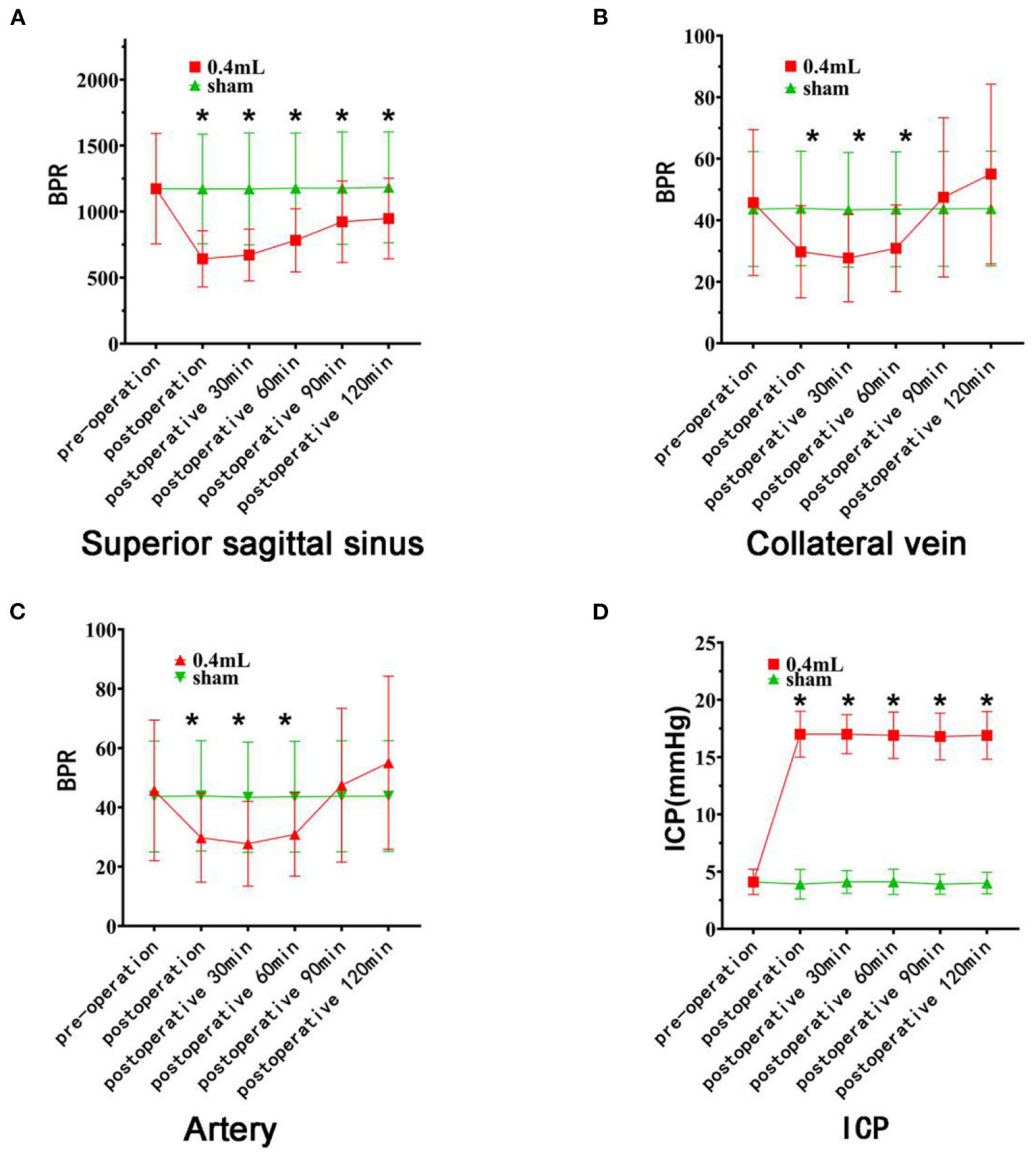
Changes in blood flow and intracranial pressure on the cerebral surface of rats. The red curve represents the 0.4 ml group, and the green curve represents the Sham group. **(A)** There was no difference in the BPR (mean ± standard deviation) of the superior sagittal sinus between the two groups before the operation. The BPR of the experimental groups decreased significantly after hematoma injection (asterisk). **(B)** There was no difference in BPR between the two groups before surgery. After hematoma injection, the BPR of the experimental group decreased significantly (asterisk) and recovered 90 min after surgery. **(C)** There was no difference in preoperative BPR between the two groups of rats. After hematoma injection, the BPR of the experimental group decreased significantly (asterisk) and recovered by 90 min after surgery. **(D)** There was no difference in intracranial pressure between the two groups before surgery, but there was a significant difference at each time point after surgery (asterisk).

In summary, the blood flow of the superior sagittal sinus, vein and artery decreased significantly from the preoperative measurement to the 60-min postoperative measurement (Figure 4).

##### Vascular BPR 60 min After Surgery

There was no significant difference in the BPRs of veins or arteries at 90 or 120 min after surgery compared with the BPRs at the same time point before surgery or the BPRs in the Sham group. The BPR of the superior sagittal sinus was significantly lower 90 and 120 min after surgery than before surgery (both *P* < 0.05).

In conclusion, the blood flow of the superior sagittal vein and artery rose and then recovered to the preoperative level within 90 min after surgery, while the blood flow of the superior sagittal sinus rose and did not recover (Figure 4).

##### Intracranial Pressure

The intracranial pressure at each time point after surgery was significantly higher than that before surgery and higher than the intracranial pressure of the Sham group at the corresponding time point (*P* < 0.05). There was no significant difference between postoperative time points (Figure 4). The blood pressure of the rats increased briefly with increasing intracranial pressure. Blood pressure gradually returns to normal baseline levels. There was no significant difference among the rats.

#### Results From the Sham Group

There was no statistical significance in the BPR of the superior sagittal sinus, vein or artery from before surgery to any time after surgery; that is, the BPR of the superior sagittal sinus, vein and artery at each time point before and after surgery remained basically stable. There was no significant difference in intracranial pressure among the time points; thus, it remained essentially stable (Figure 4).

### Blood Flow of the Acute Intraoperative Brain Bulge

**Figure 5:**
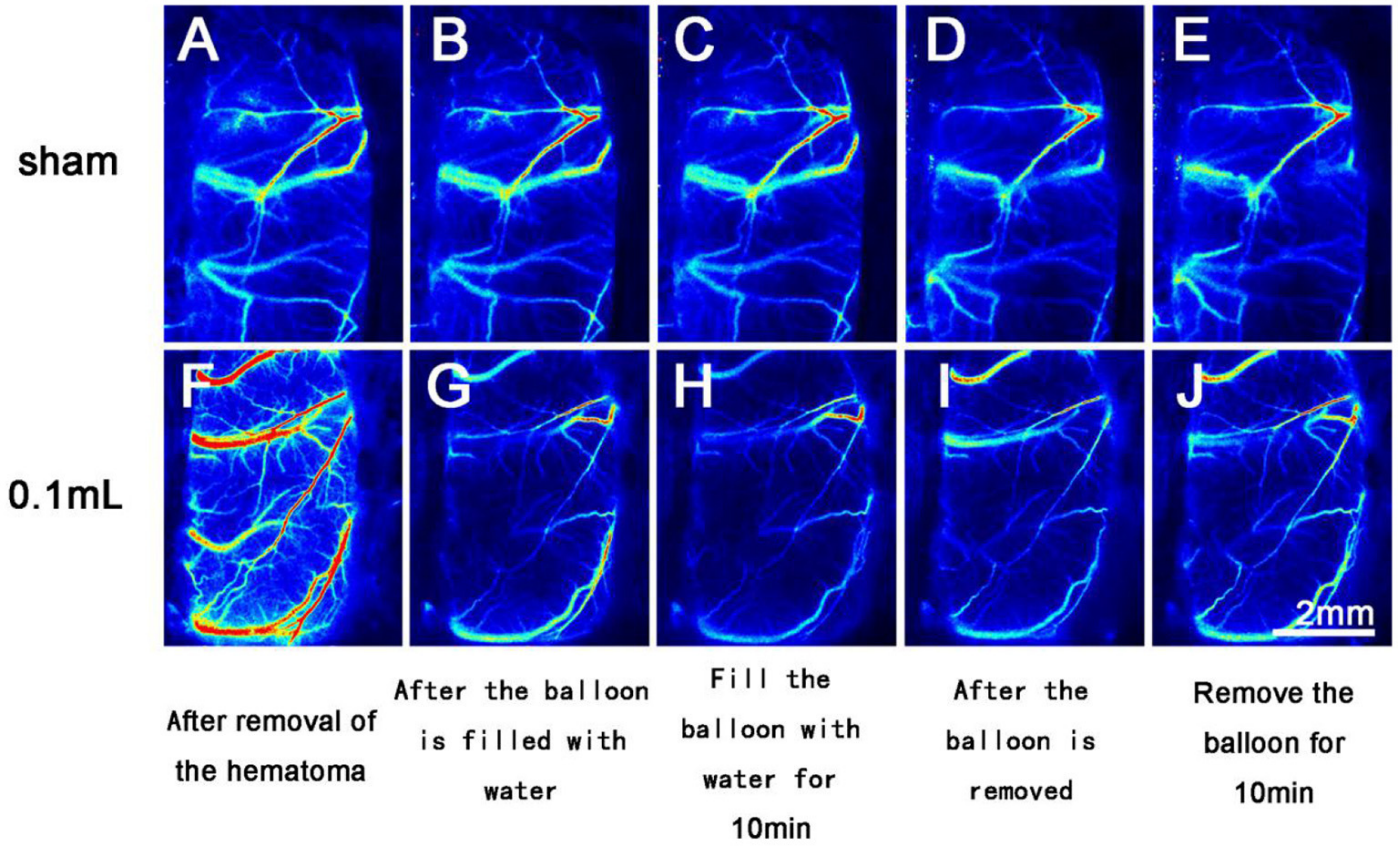
Laser speckle imaging of cerebral arterial and venous blood flow on the side with the brain bulge. **(A–E)** The cerebral surface blood flow of rats in the Sham group remained basically unchanged throughout the 40 min after hematoma removal. **(F–J)** The cerebral surface blood flow of rats in the 0.1 ml group decreased significantly after balloon filling and brain tissue swelling but gradually recovered after balloon removal.

BPR in the Sham group basically remained unchanged. In the 0.05 ml group, BPR showed a downward trend after the balloon was filled with water and an upward trend after balloon removal. In the 0.1 ml group, BPR showed a significant downward trend after the balloon was filled with water and an upward (recovering) trend after the balloon was removed (Figure 5). The changing trend of intracranial pressure in the three groups was basically the same as that of BPR.

#### Results From the 0.1 ml Group

##### Vascular BPR After Hematoma Clearance

**Figure 6:**
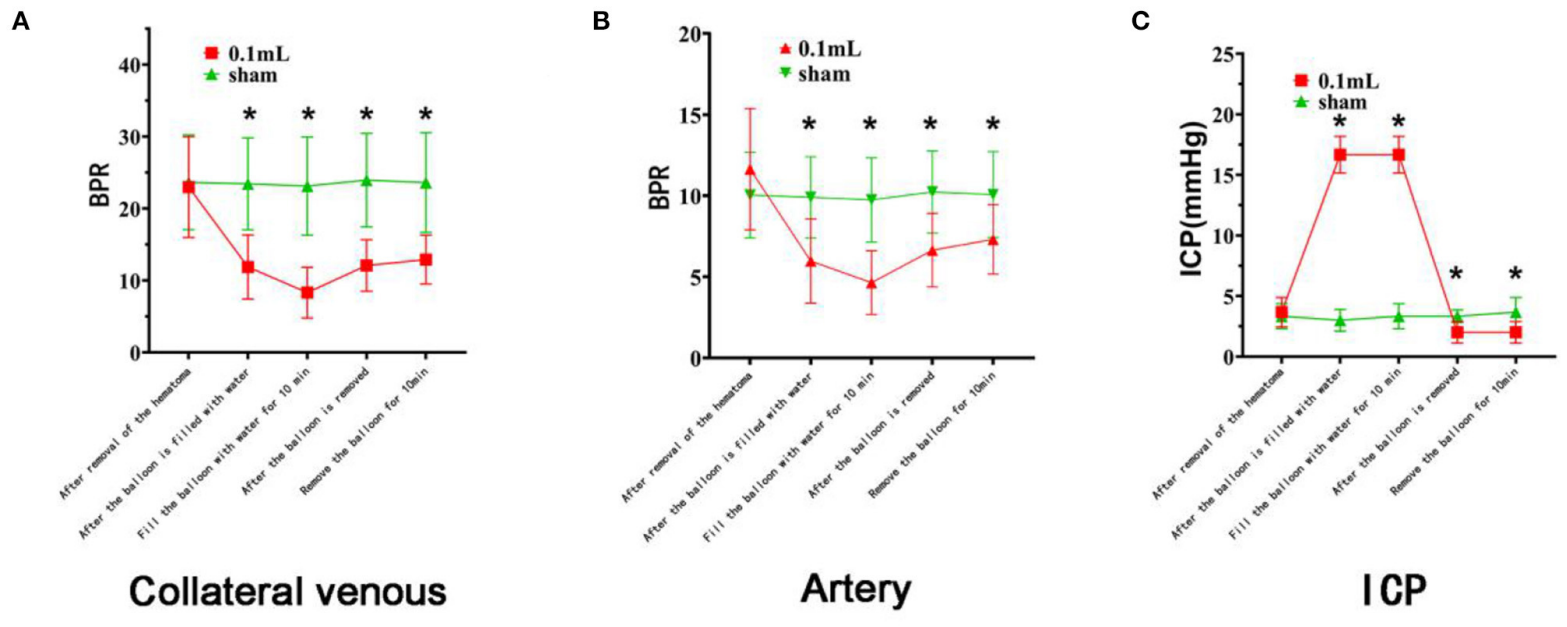
Changes in cerebral surface dynamics, venous blood flow, and intracranial pressure on the side with the brain bulge. The red curve represents the 0.1 ml group, and the green curve represents the Sham group. **(A)** There was no difference in BPR (mean ± standard deviation) between the two groups after the removal of the lateral vein hematoma. After the balloon was filled with water and the brain tissue was expanded, the BPR of the experimental groups decreased significantly (asterisk). After the balloon was removed, the BPR gradually recovered but did not return to the initial state. **(B)** There was no difference in BPR (mean ± standard deviation) between the two groups after the removal of the arterial hematomas. After the balloon was filled with water and the brain tissue expanded, the BPR in the two groups decreased significantly (asterisk). After the balloon was removed, the BPR gradually recovered but did not return to the initial state. **(C)** There was no difference in intracranial pressure between the two groups after hematoma removal, although there were significant differences at the time points after balloon filling and after balloon removal (asterisk).

The BPRs of the veins and arteries after hematoma removal were similar to those of the Sham group (Figure 6).

##### Vascular BPR After Balloon Filling

After the balloon was filled with water, the BPRs of the vein and artery were significantly lower than after the removal of the hematoma (*P* < 0.001). The BPRs of the vein and artery were significantly lower than those of the Sham group at the same time point, with *P* values of 0.000 and 0.003, respectively.

The BPRs were significantly lower 10 min after the balloon was filled with water than before surgery (both *P* < 0.001). Compared with the BPRs of the Sham group at the same time point, these were significantly reduced, with both *P* < 0.001.

In conclusion, venous and arterial blood flow decreased significantly after the balloon was filled with water (Figure 6).

##### Vascular BPR After Balloon Removal

After balloon removal, the BPRs of the veins and arteries were significantly lower than before surgery (*P* < 0.01). Compared with the BPRs 10 min after balloon filling, these values were significantly reduced (*P* < 0.05). They were also lower than the BPRs of the Sham group at the same time point, with *P* values of 0.000 and 0.004, respectively.

Ten minutes after balloon removal, the BPRs of the veins and arteries were significantly lower than before surgery (both *P* < 0.01). These BPRs were also lower than the values from 10 min after the balloon was filled with water (*P* < 0.05). Compared with the BPRs of the Sham group at the same time point, these BPRs were significantly reduced, with *P* values of 0.001 and 0.019, respectively.

In summary, after the balloon was removed, the vein and artery gradually recovered but did not recover to the initial state (Figure 6).

##### Intracranial Pressure

The intracranial pressure at each time point after balloon filling and balloon removal was significantly lower than that before surgery and lower than that of the Sham group at the same time point (*P* < 0.05). There were statistically significant differences between each time point after balloon filling and each time point after balloon removal (all *P* < 0.05). The blood pressure of the rats increased briefly with increasing intracranial pressure. Blood pressure gradually returns to normal baseline levels. There was no significant difference among the rats.

In conclusion, intracranial pressure increased significantly after balloon filling and was higher than the normal intracranial pressure. Intracranial pressure decreased significantly after balloon removal and was lower than normal (Figure 6).

#### Results From the Sham Group

There was no significant difference in the BPR of veins or arteries between the time points before and after hematoma removal and balloon filling; that is, the BPR of veins and arteries remained basically stable after hematoma removal. There was no significant difference in intracranial pressure among time points; thus, the intracranial pressure was essentially stable.

## Discussion

In recent years, cerebral circulation has been increasingly emphasized in the literature on traumatic brain injury (8, 10, 11). Researchers have found that there is a certain circulation disorder in the cerebral cortex of rats with traumatic brain injury, but the reason is not very clear; it may be vasospasm or the formation of a microthrombus (12). Moreover, ischemic changes in the cortex and certain microcirculation disorders are found in ASDH rats (13, 14). These injuries may be caused not only by the pressure of the hematoma but also by the influence of blood components (15). Other studies have indeed found that thrombin in blood and some components in blood clots are factors causing brain swelling and other serious consequences (16–18). Therefore, studying the role of cerebral circulation in ASDH will be conducive to a comprehensive understanding of the pathophysiological changes after ASDH. As acute intraoperative brain bulge is also related to cerebral circulation disorders, the study of ASDH circulation can play an important role in the study of acute intraoperative brain bulge.

In the first part of this study, blood flow on the brain surface of ASDH rats was mainly observed in the superior sagittal sinus and the arteries and veins in the hemisphere opposite the hematoma. This is because after the ASDH model was prepared, the hematoma would cover the blood vessels of the local side so that the laser could not observe the blood flow below the hematoma. In previous studies, it was found that ASDH would also affect the blood vessels covering the compressed side, resulting in decreased blood flow, which was not accounted for in those experimental studies. Therefore, the main focus of our experiment was blood flow changes in the hemisphere opposite the hematoma. The baseline BPRs in the superior sagittal sinus, vein and artery were all similar between the Sham group and the experimental group, facilitating the comparison of the BPR of each vessel at subsequent time points. As seen from the results, after the formation of ASDH, the BPRs of the veins and arteries in the superior sagittal sinus and contralateral hemisphere all decreased significantly, and the BPRs remained low until 60 min after the operation, while they recovered from 90 to 120 min after the operation. There was no significant difference between preoperative baseline and postoperative BPRs at 90 and 120 min. We conclude that ASDH can lead to a decrease in BPR in the upper sagittal sinus, cerebral surface veins and arteries in rats within a certain time, resulting in certain circulation disorders, but over time, the upper sagittal sinus, cerebral surface veins and arteries in rats can gradually compensate and recover. Previous studies in an epidural hematoma model in rats found that increased intracranial pressure led to decreased BPR of blood vessels on the brain surface, resulting in certain circulation disorders. In this study, subdural hematoma was induced in rats, and increased intracranial pressure and decreased blood flow on the brain surface were observed, consistent with previous studies. It is worth noting that subdural hematoma is very different from epidural hematoma. In an epidural hematoma, the dura is intact, and the blood does not directly contact the cortex; thus, it is simply an effect of compression. However, after subdural hematoma, there is not only a compression effect but also direct contact of blood components with the cerebral cortex, resulting in more complex effects than epidural hematoma causes. The circulatory dysfunction observed in this study may also be related to the unique cortical ischemic changes observed in subdural hematoma in rats. However, since most of the ischemic changes occurred 24 h after the formation of the hematoma, we observed the changes only in the acute stage, up to 120 min after surgery, and the circulatory function recovered thanks to the developed cerebral venous system and strong compensatory ability of rats. Over a longer observation time, more discoveries may be made.

In clinical practice, intraoperative brain bulge can lead to cerebral infarction and other complications and endanger the patient's life. The mechanisms of acute intraoperative brain bulge include late-onset intracranial hematoma, acute diffuse brain swelling, and venous reflux disorder (19). In the second part of the study, the pathomechanism of brain bulge in ASDH rats was late-onset epidural hematoma in the contralateral hemisphere (20). A late-onset hematoma can elevate intracranial pressure and cause brain tissue to be pushed out of the bone window. A refillable water balloon was placed in the epidural space of the contralateral hemisphere to simulate an epidural hematoma. When the subdural hematoma was removed, the contralateral side formed a new epidural hematoma, led by the pushing of brain tissue, and protruded through the surgically created bone window. We chose the observation time window of 40 min after hematoma removal because the rat surgical procedure is more difficult than the human operation in terms of fluid control and intraoperative care; therefore, an overly long bone flap surgery time could lead to unstable intracranial pressure in rats (21). Impact experimental indices were used to control the operation time and observation time, but even in a short time, significant changes in blood circulation were observed. Unlike the first part of the study, this part merely observed the changes in collateral veins and arteries on the side from which the bone flap was removed. To simulate a human surgical scenario as closely as possible, the bone window was limited to the size needed for removal. At the same time, simulated contralateral epidural hematoma would strongly push against brain tissue. The upper sagittal sinus will also increase the measurement error due to extrusion and compression; therefore, the blood flow was measured only in veins and arteries. The experimental results showed that venous and arterial blood flow decreased significantly after cerebral tissue swelling due to the increase in intracranial pressure and vascular circulation disorder caused by cerebral swelling. As long as the brain tissue continues to bulge, this circulation disorder cannot improve. When the cause of the brain bulge is removed in time and the intracranial pressure is reduced, the circulation disorder can be relieved, but it cannot be restored to the pre-bulge state in a short time.

Although the present study found significant cerebral circulation disorders associated with acute intraoperative brain bulge, the causal relationship between these disorders and brain bulge remains unclear. It may be that the interaction between the two forms a vicious cycle. Cerebral circulation disorder after craniocerebral injury leads to acute intraoperative brain bulge, which further aggravates any cerebral circulation disorder. This may be a new angle from which we can clarify the genesis and development mechanism of brain bulge.

Brain swelling is prone to occur after ASDH, and such swelling has little to do with the thickness of the hematoma. Even when the hematoma is thin, swelling can occur, indicating that the key reason for such swelling is not simple compression by the hematoma and the high intracranial pressure caused by the compression (22), and such swelling amounts to an acute brain bulge during surgery. Another study found that a large cerebrospinal fluid (CSF) metastasis can cause rapid swelling of brain tissue (23), indicating that the change in tissue fluid composition is likely to be the key factor of acute intraoperative brain bulge. After cerebrospinal fluid, the main fluid in the brain is blood. The hypothesis that diffuse brain swelling causes acute intraoperative brain bulge has led to the proposal of dysfunction of vascular regulation (6, 24, 25), which suggests that circulation problems need to be considered in the occurrence and development of intraoperative brain bulge. However, there is insufficient evidence for this theory, and it is the only mechanistic description that has been presented in the literature. In previous studies on brain bulge, a factor that has been mentioned without an in-depth explanation is venous blood flow (2, 19). In many cases of traumatic acute subdural hematoma, the surface of the brain is found to collapse when the hematoma is removed, but the brain tissue progressively expands after a few minutes. However, in many cases, secondary intracranial hematoma can be excluded, indicating that there is a certain factor that can quickly cause brain tissue swelling, but the progression of brain edema is usually not fast, and it may be that vascular circulation disorders play a role. In the blood circulation, the venous system carries the largest volume of blood; therefore, dysregulated venous circulation is likely to be an important mechanism of acute intraoperative brain bulge.

In summary, this study found that subdural hematoma had a certain impact on cerebral blood circulation, for example, in the veins and arteries; after the removal of the hematoma, brain tissue swelling appeared, and the circulation disorders were aggravated. Timely removal of the causes of acute intraoperative brain bulge can reduce intracranial pressure, which can alleviate circulation disorders. If the results of the rat experiment translate to the treatment of humans, these results suggest that clinicians should identify the cause of intraoperative brain bulge as soon as possible and treat it, which would facilitate the recovery of patients' cerebral circulation, reduce the occurrence of cerebral infarction and other complications, and reduce the rates of disability and mortality among patients.

Recent clinical studies suggest that in order to effectively prevent and treat acute intraoperative brain bulge, it is necessary to comprehensively evaluate the patient's condition quickly and take effective measures (26). First, the timing of surgery should be rationally and individually selected according to CT examination (27, 28). In this way, the beginnings of the intraoperative bulge can be prevented more effectively, especially in patients with complex and severe injuries (29). Second, according to the understanding of delayed intracranial hematomas, the concept of stepwise decompression is increasingly recognized as important, enabling improvements in all varieties of brain trauma surgery, such as drilling drainage with removal of the hematoma (30), dura mater incision decompression (31), step-by-step dura mater incision combined with contralateral open-brain surgery (32), and others. The core idea is slowly to reduce intracranial pressure and reduce secondary bleeding caused by rapid removal of compression factors. In clinical research and practice, step decompression reduces the incidence of prolapse and has good therapeutic benefits (33, 34). Continuous monitoring of intraventricular pressure, appropriate external ventricular drainage, and rational use of mannitol can prevent or effectively treat swelling (35). This comprehensive strategy for the prevention and treatment of acute brain bulge significantly improves the success rate of treatment and the prognosis of patients (25, 36). Although these therapeutic strategies have been proposed, acute intraoperative brain bulge cannot be completely avoided, and many conditions cannot be explained by current theories. Therefore, through these laboratory experiments, we hope to provide a basis for the development of animal models of brain bulge and provide new ideas for exploring the occurrence, development mechanism and treatment strategies of acute intraoperative brain bulge to help improve our understanding of acute intraoperative brain bulge as soon as possible.

## Conclusion

After acute subdural hematoma, the intracranial pressure of rats increased, and the blood flow of the veins and arteries of the superior sagittal sinus and the brain surface on the side opposite the hematoma decreased significantly for a certain time. The veins and arteries recovered compensatively 90 min after the formation of the hematoma, but the superior sagittal sinus did not recover. Acute subdural hematoma may cause both high intracranial pressure and impaired blood circulation to the brain, which may be a factor in secondary damage. Based on the simulation of brain bulge caused by delayed intracranial hematoma, venous and arterial blood flow decreased significantly when brain tissue was expanded. If brain tissue continues to bulge, this circulatory disorder can persist. When the cause of brain bulge is removed in time and the intracranial pressure is reduced, the circulation disorder can be relieved, but it cannot be restored to the same state as before the brain bulge in a short time. Clinicians should take note of the cerebral circulation problems of patients with craniocerebral injury before beginning surgery. If the cause of brain bulge is late-onset intracranial hematoma, clinicians should perform surgery in a timely manner to remove the hematoma, relieve circulation disorders, and prevent more serious complications.

## Data Availability Statement

The raw data supporting the conclusions of this article will be made available by the authors, without undue reservation.

## Ethics Statement

The animal study was reviewed and approved by 900 Hospital Ethics Committee.

## Author Contributions

LX and CW performed data acquisition, analyses, and manuscript writing. LW performed statistical analysis and contributed to the manuscript. SW supervised the whole project. All authors made substantial contributions to conception and design, analyses and interpretation of data, revising the article, read the manuscript, and approved its submission.

## Funding

This work was supported by Fujian Provincial Science and Technology Innovation Joint Fund Project (2019Y9045).

## Conflict of Interest

The authors declare that the research was conducted in the absence of any commercial or financial relationships that could be construed as a potential conflict of interest.

## Publisher's Note

All claims expressed in this article are solely those of the authors and do not necessarily represent those of their affiliated organizations, or those of the publisher, the editors and the reviewers. Any product that may be evaluated in this article, or claim that may be made by its manufacturer, is not guaranteed or endorsed by the publisher.
